# The Characteristics of Sentinel Lymph Node Biopsy in Cutaneous Melanoma and the Particularities for Elderly Patients—Experience of a Single Clinic

**DOI:** 10.3390/diagnostics13050926

**Published:** 2023-03-01

**Authors:** Florin Bobircă, Tiberiu Tebeică, Adela Pumnea, Dan Dumitrescu, Cristina Alexandru, Laura Banciu, Ionela Loredana Popa, Anca Bobircă, Mihaela Leventer, Traian Pătrașcu

**Affiliations:** 1 Surgery Department, Carol Davila University of Medicine and Pharmacy, 050474 Bucharest, Romania; 2 Surgery Department, Dr. Ion Cantacuzino Clinical Hospital, 011437 Bucharest, Romania; 3 Dr. Leventer Centre, 011216 Bucharest, Romania; 4 Gauss Clinics, 011255 Bucharest, Romania; 5 Internal Medicine and Rheumatology Department, Dr. Ion Cantacuzino Clinical Hospital, 011437 Bucharest, Romania; 6 Marie Curie Emergency Children’s Hospital, 041451 Bucharest, Romania; 7 Internal Medicine and Rheumatology Department, Carol Davila University of Medicine and Pharmacy, 050474 Bucharest, Romania

**Keywords:** melanoma, sentinel lymph node biopsy, elderly

## Abstract

Background: Melanoma is a malignant tumor that determines approximately 80% of deaths as skin cancer-related. The sentinel lymph node (SLN) represents the first filter of tumor cells toward systemic dissemination. The primary objective was to outline the surgical specifics of the sentinel lymph node biopsy (SLNB) technique, correlate the location of the lymph node with the radiotracer load, and identify the characteristics of older patients. Methods: In this prospective study, 122 cases of malignant melanoma needing SLNB technique were included, between June 2019 and November 2022, resulting in 162 lymph nodes removed. Results: Patients’ mean age was 54.3 ± 14.4 years old, the prevalence of 70 years and older being 20.5%. The rate of positive SLN was 24.6%, with a single drainage in 68.9% of cases. The frequency of seroma was 14.8%, while reintervention 1.6%. The inguinal nodes had the highest preoperative radiotracer load (*p* = 0.015). Patients 70 years old or older had significantly more advanced-stage melanoma (68.0% vs. 45.4%, *p* = 0.044, OR = 2.56) and a higher rate of positive SLN (40.0% vs. 20.6%, *p* = 0.045,OR = 2.57). Melanoma of the head and neck was more common among older individuals (32.0% vs. 9.3%, *p* = 0.007,OR = 4.60). Conclusions: The SLNB has a low rate of surgical complications and the positivity of SLN is not related to radiotracer load. Elderly patients are at risk for head and neck melanoma, have more advanced stages, a higher SLN positivity, and a greater rate of surgical complications.

## 1. Introduction

Melanoma is a malignant tumor originating in the melanocytic cells of the skin, and less frequently in the melanocytes of the eyes, mucous membranes, and meninges [1]. Although it represents approximately 1% of all skin tumors, melanoma determines approximately 80% of deaths caused by skin cancer [2]. According to European Cancer Information System, it is estimated that cutaneous melanoma represents 4% of all new cancer diagnosed in 2020 (excluding non-melanoma skin tumors) and 1.3% of all deaths caused by cancer [3].

Considering the increasing trend in the incidence of melanoma at all ages, in the next decade, the implementation of effective methods of prevention and early detection of the disease will be mandatory. Furthermore, it is well known that, not just for melanoma, but for all types of cancer, early initiation of therapy in advanced patients minimizes mortality and morbidity [4,5]. Disease staging, according to the eighth edition of the American Joint Committee on Cancer, followed by appropriate management are important factors influencing the survival rate of melanoma patients [6,7].

In the metastasis process, the primary site affected by cutaneous melanoma is represented by the regional lymph nodes, their evaluation to identify macrometastases detected clinically/imaging or micrometastases by sentinel lymph node biopsy (SLNB) is the most important prognostic factor in the early stages of the disease [8].

**Figure 1:**
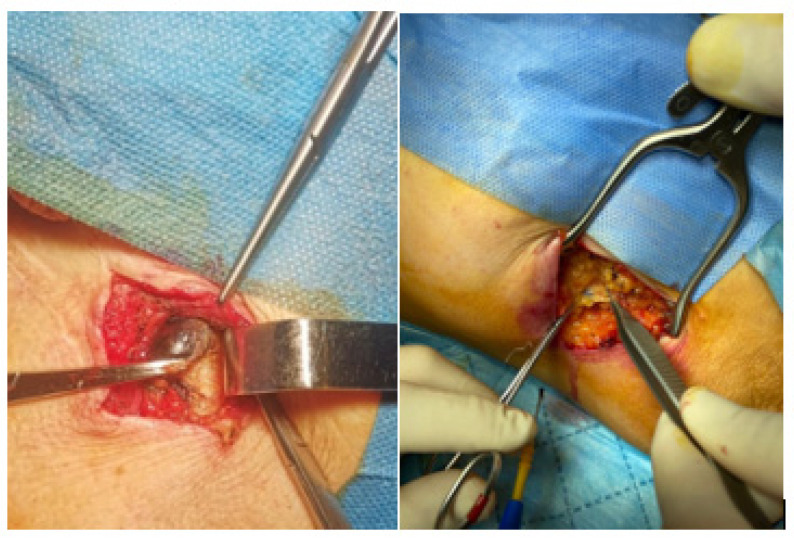
Sentinel lymph node—surgical specimen—Dr. Leventer Centre Collection.

The sentinel lymph node (SLN) represents the first drainage station of the primary tumor, therefore, the first filter of tumor cells toward systemic dissemination [9]. In the past, regional lymph node dissection (Figure 1) was used to confirm the presence of local lymph node metastases, burdened by long-term and short-term complications. Based on the model of lymphatic extension of tumor cells, in the 1990s, Morton et al. developed the sentinel node biopsy, a less invasive technique for evaluating the condition of local lymph nodes. The sentinel lymph node biopsy has evolved into a staging technique for patients without clinically detected node metastases or through imaging. In this manner, unnecessary nodal dissections, burdened by high morbidity, are avoided, especially for those patients with multiple comorbidities such as diabetes mellitus and cardiovascular disease [1,10]. As a result, SLNB has been approved as a technique and included in the TNM (tumor, node, metastasis) staging classification for cutaneous melanoma [11].

According to the ASCO-SSO (American Society of Clinical Oncology—Society of Surgical Oncology) guideline for SLNB in melanoma, and ESMO (European Society for Medical Oncology), sentinel lymph node biopsy is recommended for patients in stage IB/II of the disease with the following criteria:

Thin melanomas: 0.8 to 1.0 mm Breslow thickness with or without ulceration or <0.8 mm Breslow thickness with ulceration (T1b stage) after a thorough discussion with the patient of the potential benefits and risk of harms associated with the procedure (should be considered).

Melanomas with intermediate thickness: Breslow thickness of >1.0 to 4.0 mm (T2/T3 stages) (should be recommended).

Thick melanomas: 4.0 mm in Breslow thickness (T4 stage) after a thorough discussion with the patient of the potential benefits and risk of harms associated with the procedure (should be considered) [7].

The sentinel lymph node biopsy is necessary when it is impossible to acquire the tumor thickness after a superficial biopsy or after the lesion has undergone previous cryotherapy or electrodesiccation [12].

**Figure 2:**
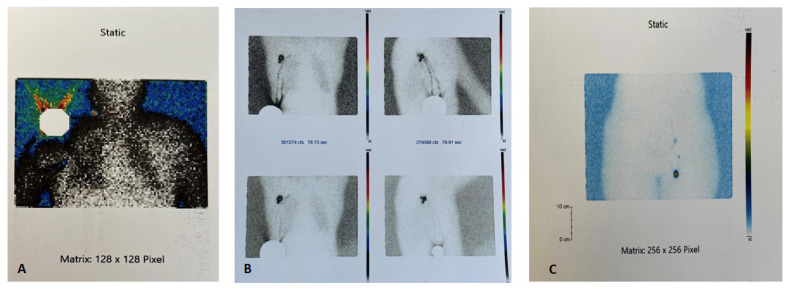
Lymphoscintigraphy aspect—Dr. Leventer Centre Collection. (**A**). Radiotracer capture on melanoma site (right shoulder). (**B**). Axillary sentinel lymph node. (**C**). Femoral sentinel lymph node.

Over time, sentinel lymph node biopsy, initially associated with complete lymph node dissection, has been studied and applied in the case of skin and mucous tumors of the head and neck. The biopsy of the sentinel node of melanomas in these cases was controversial due to the complex architecture of the lymphatics at the head and neck level. The main concern was the multitude of lymph nodes that drain the primary tumor from the head and neck, causing less predictable lymphatic drainage, thus a change in the accuracy of SLNB [13]. Currently, this can be corrected by using imaging techniques (Figure 2) such as cross-sectional X-ray computed tomography (CT) and single photon emission computed tomography (SPECT) to accurately detect the radiotraced lymph node [14]. Another disadvantage of this topography is the presence of numerous vital vascular structures and cranial nerves which may compromise the procedure’s safety [13].

Another important issue regarding the particularities of SLNB is related to the patient’s age, as it is known that melanoma is frequently diagnosed in the elderly. In the age group 70 and older, the rate of positive SLN is higher than in the rest of the population, head, and neck site of the melanoma is the region most frequently affected, and the tumors are usually in advanced stages (T3/T4). Only after the positivity of the SLN, did the oncologists take into account the immune therapy, considered escaped therapy, and graft multiple possible complications [15,16].

The elderly should be the main focus of secondary melanoma prevention, that is, early diagnosis and screening to reduce mortality. Older people are more likely to develop and die from melanoma. The elderly may also have fewer treatment options because they may be less able to endure drug side effects, are more likely to have drug interactions, or may be excluded from clinical trials due to age eligibility requirements [17].

Sentinel lymph node biopsy represents the standard of care in the management of early stage melanoma patients. According to NCCN, depending on the subclinical micrometastatic disease in the SLN, 5–40% of patients who perform SLNB will be upstaged to pathologic stage III. Considering these, the patient must be informed about future management options, including imaging tests, adjuvant therapy, clinical trial enrollment, the requirement for a complete lymph node dissection (CLND), and routine follow-up [18,19,20,21,22].

The main endpoint of this study was to monitor the surgical particularities of the sentinel lymph node technique among melanoma patients. Secondary endpoints included the correlation of the lymph node’s location with the radiotracer load, with positivity, and the identification of significant differences related to the diagnosis of melanoma at an advanced age (70 years and older).

## 2. Materials and Methods

We conducted a longitudinal, prospective cohort study that involved the follow-up of 122 cases of malignant melanoma with an indication for the sentinel lymph node technique who underwent surgery in a private dermatology clinic in Bucharest (Dr. Leventer Centre) between June 2019 and November 2022, resulting in a total of 162 lymph nodes.

Inclusion criteria were patients older than 18 years old, diagnosis of melanoma with Breslow index >= 0.8 mm, lack of lymph node(s) or organ metastatic involvement (clinical and imaging), and patients within <= 6 weeks of diagnosis by excisional biopsy.

Exclusion criteria were as follows: patients < 18 years old, Breslow index < 0.8 mm, contraindication of surgery as a result of the pre-anesthetic evaluation, palpable peripheral lymph node(s), organ metastases (MRI or CT scan) and patients that had no detection of sentinel lymph nodes on lymphoscintigraphy. All patients gave written informed consent and ethical approval was obtained.

Surgery was performed within an interval of up to 6 h from lymphoscintigraphy in all patients.

The surgical strategy needed an interdisciplinary operative team, which included, in addition to the oncological surgeon, the plastic and the buco-maxillo-facial surgeon, as determined by the location of the melanoma, according to Breslow, the outcome of the preoperative lymphoscintigraphy, the indication connected to the oncological safety margins (1 or 2 cm) and the patient’s request. All patients received the combination strategy of detecting the sentinel lymph node with Tc^99^ and vital dye injection (methylene blue).

Demographic data were registered using a questionnaire fulfilled by all patients. Monitored variables were the following: location of melanomas, and lymph node(s) surgically removed, positivity of the lymph node(s) (positive or negative, IHC testing), BRAF gene mutation, Breslow index, tumor stage, tumor location, serum S100 protein testing, interval (days) between diagnostic excisional biopsy and performing the sentinel lymph node technique, issues related to lymphoscintigraphy (number of draining lymphatic basins, number of sentinel nodes, radioactive load at melanoma’s scaring site, but also preoperative radiotracer load and intraoperative and ex vivo sentinel lymph node radiotracer load). Additionally, data regarding the presence of personal cardio-vascular history, use of anticoagulant or antiaggregant therapy, oncological history, type of anesthesia, pre- and postoperative prophylactic antibiotic therapy, duration of surgery—node + excision with safety margins (cm), reinterventions, intraoperative complications, appearance of seromas, duration (days) until wound healing were monitored.

### Sentinel Lymph Node Surgical Technique

The sentinel lymph node biopsy is a minimally invasive surgical technique that aims to detect and remove the first lymph node(s), from the lymphatic basin(s) that drains the melanoma area. Prior to the re-excision with oncological margins of the scar, SLNB is completed within 4–6 weeks after the excisional biopsy [18].

A radiotracer, 0.5–1.0 mCi radio-colloidal Technetium Tc-99m, is injected intradermally in 4–5 spots surrounding the scar, at a maximum distance of 1 cm from this, preoperatively, on the day of the intervention or 24 h before. The nuclear medicine department performs lymphoscintigraphy as the following step to identify lymphatic drainage. Additional images obtained using single photon emission computed tomography (SPECT) may be necessary for certain anatomical regions, such as the cephalic extremity or the pelvic region, to identify the sentinel lymph node [23,24]. Methylene blue can be injected intradermally at the level of the scar to increase the method’s sensitivity. The gamma probe detects radioactivity and blue staining serves to identify the sentinel node properly. After identifying the sentinel node’s high radioactivity regions with a gamma probe, small incisions are made oriented in the direction of the nodes’ further dissection. The sentinel node is located, excised, and the gamma probe is used to analyze the radioactivity ex vivo. Lymph node exploration is continued to identify the presence of other drainage stations by identifying nodes with radioactivity greater than 10% of the initially identified node [25].

Statistical analyses were performed using SPSS Statistics version 20. Nominal variables were reported as frequency and percentage, while comparison was performed using chi-squared test and Fisher’s test. Continuous data were presented as mean ± standard deviation (SD) and median (minimum–maximum), respectively, while differences between groups were established using Mann–Whitney U test and Kruskal–Wallis test. Odds ratio and 95% confidence interval were calculated using binary logistic regression. A *p*-value < 0.05 was statistically significant for all tests.

## 3. Results

The total number of patients who underwent surgery was 122, with a total of 162 lymph nodes removed. The general characteristics of patients enrolled are illustrated in Table 1.

The patients’ mean age was 54.3 ± 14.4 years old (y.o), ranging from 21 to 84 y.o, with a prevalence of 70 years old and older of 20.5% (N = 25). The majority of patients had just one lymph node removed (84, or 68.9%), while two lymph nodes were for 29.5% of patients (N = 36) and three for 1.6% of the cohort. The frequency of positive SLN patients was 24.6%, there were three patients with two positive lymph nodes.

In terms of gender, the rate of men was 50.8% (N = 62). Furthermore, 61.6% of women were at menopause (N = 37 from 60).

The most common region of melanomas was the thorax (39 patients—32.0%), followed by lower limbs (27 patients—22.1%), upper limbs (21 patients—17.2%), abdomen (18 patients—14.8%), and head and neck areas (17 patients—13.9%).

According to NCCN criteria for tumor staging, it was found that most melanomas were classified as PT1b-30 (24.6%), followed by pT2a-26 (21.3%), pT4b-24 (19.7%), pT3b-20 (16.4%), pT3a-10(8.2%), pT4a-7(5.7%), pT2b-4 (3.3%), and pT1a-1(0.8%), respectively. Therefore, advanced tumor stages pT3 and pT4 accounted for half of the biopsied melanomas.

The Breslow index had a mean of 3.04 ± 2.8 and it was used to determine the oncological safety margins, which were as follows: 1 cm for 35 patients, 1.5 cm for 3 patients (2.5%), and 2 cm for the vast majority of the population (68.9%), respectively.

The period from the tumor biopsy to the excisional surgery with sentinel lymph node removal had a mean of 30.3 ± 5.1 days.

The BRAF gene tested positive in 22 of the 31 examined cases, but for 2 individuals no data regarding BRAF gene screening was registered. Serum protein S100 should be checked particularly in patients with pT3 or pT4 tumor stages. Serum protein S100 was tested in 8 of 122 individuals in our sample and it was only positive in 1—a stage pT4b patient with a positive BRAF gene. Only 5 of the 122 patients had a history of cancer, and the mean body mass index was 24.1 ± 2.7 kg/m^2^.

General anesthesia was necessary for the majority of the surgeries (75.4%), however, local anesthesia was helpful in 30 cases (25.6%).

Despite the fact that a third had cardiovascular diseases (31.96%), the complications of general anesthesia during surgery were only seen in three patients, including two with cardiac rhythm abnormalities and one with desaturation. Six patients (4.9%) with a cardiovascular history required switching to fractionated heparin therapy and two of them also required reintervention for bleeding. As for intraoperative surgical complications, we specifically emphasize three cases with small arterial injuries that required extra hemostasis, as well as one case of nerve damage that had no neurological consequences. Thus, a total of seven cases (5.7%) had intraoperative complications, the surgery’s duration for those individuals being slightly longer, but no significant difference was registered when compared with the rest of the sample studied (130.0 ± 32.3 vs. 124.7 ± 27.8, *p* = 0.821).

The entire cohort had a mean of the procedure’s duration of 125.0 ± 27.9 min. Patients with more than one lymph node removed had a statistically significant longer surgery compared to those having only one lymph node (153.9 ± 28.4 vs. 111.9 ± 14.9, *p* < 0.001). The tumor’s location was another variable that affected how long the procedure took; head and neck melanomas required a more complex and time-consuming procedure (145.9 ± 28.8 vs. 121.6 ± 26.5 min, *p* < 0.001).

No patient had postoperative infectious complications, and almost all patients (119–97.54%) received prophylactic antibiotic therapy preoperatively (single dose), but also postoperatively (mean duration 3.8 ± 1.4 days). It should be noted that the three patients who did not receive antibiotic therapy had a history of drug polyallergies.

The duration of wound healing at the melanoma site had a mean of 12.5 ± 1.9 days, ranging from 10 to 21 days.

In terms of long-term surgical complications, in this study, we found that the prevalence of seroma was 14.8%. Comparing head and neck melanomas with melanomas in other regions, this complication was only observed in cases involving lymph node removal from the axillary and inguinal basins, not for cervical areas (*p* = 0.073). Percutaneous puncture with ultrasound guidance was performed for all patients with seroma (3–4 repeated punctures).

In contrast to other regions, patients with head and neck melanoma were older (61.6 ± 16.9 vs. 53.1 ± 13.6 y.o, *p* = 0.022) and had a greater Breslow index (4.0 ± 3.9 vs. 2.8 ± 2.5, *p* = 0.257), but a lower rate of positive SLN (17.6% vs. 25.7%, *p* = 0.474).

Characteristics of lymph nodes are shown in Table 2. Out of the 162 removed nodes, the prevalence of positive sentinel lymph node was 20.4%. The lymph distribution was as follows: 83 in the axillary region, 40 in the inguinal region, and 39 in the cervical region. There were 4 positive sentinel nodes from cervical region, 19 from axillary, and 10 from the inguinal area. At the site of the node excision, the axillary area exhibited the longest mean time for scarring, 7.73 ± 0.5 days.

Regarding lymphoscintigraphy, the detected radioactivity was compared depending on the location of the lymph nodes. Radioactivity was registered at the melanoma’s scar site, but also before surgery, during surgery, and ex vivo for the excised lymph node(s). Patients with higher levels of reactivity at the melanoma site, as well as during surgery and ex vivo had their reactive nodes excised from the inguinal regions, ex vivo registration being notably different from the cervical and axillar region. Preparatory nodes’ radioactivity varied depending on the area, with inguinal nodes having a significantly higher median of radioactivity than the rest, *p* = 0.015. There were no statistically significant results when the node radioactivity reported was compared between positive and negative SLN.

**Table 2:** Characteristics of lymph nodes.

	N = 162	Cervical N = 39	Axillar N = 83	Inguinal N = 40	*p*-Value
Positive SLN	33, 20.4%	4, 10.3%	19, 22.9%	10, 25.0%	0.191
Scar (melanoma)	10,000.0	17,700.0	18,500.0	18,000.00	0.521
Median (min,max)	(1900–30,000)	(1900–30,000)	(2200–30,000)	(3000–30,000)	
Preoperative	655.0	760.0	550.0	783.0	0.015
Median (min,max)	(20–18,000)	(90–18,000)	(20–2700)	(46–2800)	
During surgery	1335.0	1200.0	1200.0	1700.0	0.184
Median (min,max)	(60–12,000)	(220–7500)	(60–12,000)	(120–7050)	
Ex vivo	1500.0	1000.0	1500.0	1600	0.030
Median (min,max)	(50–9100)	(130–7600)	(50–9100)	(140–6900)	
Ganglion site healing period (days) mean ± SD	7.6 ± 0.6	7.4 ± 0.6	7.73 ± 0.5	7.6 ± 0.6	0.014

Characteristics of patients 70 years old or older

In our study, there were 25 patients 70 years old or older; the comparison between this subgroup with the rest of our cohort is shown in Table 3. Advanced stages of melanoma were more frequent among older patients, 68.0% vs. 45.4%, *p* = 0.044, OR = 2.56 (1.00–6.49). A positive sentinel lymph node was found in 40.0% of cases among 70 y.o or older patients, while only 20.6% in younger patients, with a borderline statistical difference, *p* = 0.045, OR = 2.57 (1.00–6.56).

Another noteworthy observation is that melanoma of the head and neck is more common among individuals aged 70 y.o and older, with a frequency of 32.0% vs. 9.3%, a statistically significant difference, *p* = 0.007, OR = 4.60 (1.55–13.61). There was no significant difference in the number of lymph nodes surgically removed following lymphoscintigraphy, with the majority of patients in both subgroups having only one lymph node removed (68.0% vs. 69.1%).

Older subjects had more cardiovascular diseases, such as hypertension, atrial fibrillation, and a history of myocardial infarction (68.0% vs. 22.7%, *p* =< 0.001). Additionally, out of six patients, five required switching anticoagulant therapy (*p* = 0.001). The only two cases of reintervention due to hemorrhage are in older patients, who both had a history of atrial fibrillation and needed anticoagulant switching.

Regarding the intraoperative complications, from a total of seven cases, five were from patients 70 y.o or older, *p* = 0.004, OR = 11.87 (2.15–65.60). Older individuals in this cohort required longer surgeries, with a mean of 131.6 ± 31.4 min compared with younger ones, 123.30 ± 26.9 min (*p* = 0.082).

Patients 70 y.o or older were more likely to develop post-operative seromas (24.0% vs. 12.4%, *p* = 0.202), and needed significantly more time to heal (*p* = 0.004, OR = 1.38 (1.08–1.75))

## 4. Discussion

According to our data, this is the first Romanian complex study on the use of the sentinel lymph node technique in malignant melanoma, as a key factor of diagnosis for the disease stage, which brings additional information about patients diagnosed with cutaneous melanoma. There is no national data to indicate the prevalence of melanoma in our country and the use of the sentinel ganglion technique in daily practice is subliminal, but still increasing compared to previous years. The study published in 2019, by the Dutch team, rigorously highlights, along with the prevalence of melanoma at the national level, the upward trend (statistically validated) of increasing the use of the sentinel ganglion technique among patients with primary melanoma [26].

The average age of the patients enrolled in our study was 54.3 ± 14.4 years, and the proportion of male patients was 48.8%, results being absolutely superimposed with the data from a Spanish study published in 2014 on a comparable population in terms of number of patients, in which the average age was 55.6 ± 15 years, and the percentage of male patients was 50.8% [27].

Regarding the location of the melanoma in our study, the thorax was where the melanoma was most frequently identified, followed by lower limbs, upper limbs, and head and neck site (46,8%, 22.1%, 17.2%, 13.9%, respectively). In a Spanish study, the distribution was similar regarding the trunk and upper limbs and completely different regarding the head and neck region, registering a much lower frequency of 2.4% (three patients). The majority of patients had one lymph node removed (67.3%), two nodes for 29.5% of patients and three nodes for 1.6%, while in the Spanish group, the authors identified single drainage for 78.4% versus 21.6% multiple drainages [27]. According to another study, a Bulgarian one, it was found that approximately 70% of melanoma cases had a single sentinel lymph node excised, and for the rest, two or more lymph nodes were removed [28].

The positivity rate in the group from our clinic was 24.6%. The data published in 2016 by the work team led by Leiter showed that in a larger German cohort, the sentinel lymph node positivity rate was very similar to ours, 23%, the population being followed for a longer period, approximately 9 years (2006–2014) [29].

Regarding the Breslow index, in our study the average value was 3.04 ± 2.8, while in a study analyzing the characteristics of 1663 patients with melanoma, the Breslow index had an average value of 1.34 ± 2.24, the significant difference being probably due to the large number of patients, but also to the early diagnosis of melanoma in the Austrian study [30].

The safety margins are in accordance with the international recommendations, respectively, 1 or 2 cm, in line with the Breslow index, and the closure of the resulting integumentary defects was performed by the plastic surgeon [31,32].

The sentinel lymph node technique is grafted by minimal postoperative complications versus total lymphadenectomy which is associated with high morbidity, both perioperatively and after, as it is shown by the team led by Leiter in a study published in 2016. Adverse events included lymphoedema, lymph fistula, seroma, and infection [29].

The data resulting from our study highlight a limited number of postoperative complications, 1.6% reinterventions for bleeding, and 14.8% of patients who developed seromas. None of the seromas required surgical drainage, all were evacuated percutaneously under ultrasound guidance. In comparison, in a study published in 2019, postoperative complications were found in a number of 39 patients, respectively, 9.5%, and were represented by: wound infection in 24 (5.9%), seroma and lymphorrhea in 15 (3.7 %), wound dehiscence in 7 (1.7%), lymphocele in 6 (1.5%), and others in 3 (0.7%) [33].

On the other hand, in our paper, during surgery, there were a number of seven cases that had complications related to bleeding or nerve damage and to anesthesia (rhythm disorders, desaturations). The duration of the surgery in this study was on average 125.0 ± 27.9 min, it includes both the detection and excision of the sentinel lymph node, as well as the excision with safety margins and also the coverage of the remaining skin defect. Furthermore, the rate of postoperative complications was not correlated with the duration of the surgery, an issue also confirmed by another research [33].

When dividing the cohort by the age limit—70 years and older—, 20.5% belonged to the elderly group. Using the same age cutoff, a French paper identified 30% of the population as elderly [15].

Statistically, it was demonstrated that the head and neck region was the most likely area for melanoma in patients above the age of 70 (by comparing with the features of individuals under the age of 70). Additionally, this population is at risk for developing advanced cancer stages; in our study, more than half of elderly patients had stage pT3 or pT4 melanoma. Our findings are consistent with the French study (1621 patients), which was conducted more than 15 years ago and on a much bigger scale and revealed that 36.7% of senior people had advanced stages of the disease and that the head and neck region was the site where it occurred most frequently (29.4%, *p*  < 0.001) [15]. Additionally, an American study, published in 2013 and addressed to elderly patients and their particularities, showed that patients over 70 years old (25.5%), diagnosed with melanoma, are the ones with a more advanced stage of the disease (*p* = 0.001) and higher Breslow index (*p* = 0.010) [34].

Moreover, the rate of positive sentinel nodes in this paper among 70 y.o or older was significantly higher than for younger patients (40.0%), a result in line with a more recent study presented at the American Dermatology Conference in 2022, which demonstrated that the elderly population has a higher degree of sentinel node positivity 29.3% versus 18.3% among those younger, in their case the age cut-off being 75 years old [35].

As is well established, our analyses also showed that older patients have more frequent cardiovascular comorbidity (31.9%), as well as requiring re-interventions for postoperative complications more often. As a result, this group also included the majority of patients who received preoperative anticoagulation. In the study conducted by Fleming et al., cardiovascular pathology was more frequent in the elderly group (85%), but there are no data described in relation to anticoagulant therapy or postoperative complications [34]. The data from the Danish registry showed a lower rate of comorbidities associated (predominantly cardiovascular) of 19% with just half of these accounting for only one underlying pathology [36].

In this paper, the group of older patients had a significantly longer period of wound healing. Additionally, in elderly patients, the BRAF gene was detected in six cases of a total of nine patients 70 y.o or older who were tested.

Regarding primary head and neck melanoma, the frequency in the studied population was 13.9%, while data from Serbia released in 2020 indicated a percentage of 14.99% for this location. Despite the Serbian study’s longer study period of 10 years (2005–2015) compared to our study’s shorter term of three and a half years, these numbers were comparable [37]. In a recent study on a bigger cohort, the frequency of melanoma of the head and neck was similar to ours, 12%, but the overall positivity of SLNB was less frequent in comparison to our results, only 6.7% [38].

Considering that the head and neck group was more frequent in our population [39] than the results from other countries, we set out to identify the particularities of those individuals. In this subgroup, patients were older, the Breslow index had a greater value; meanwhile, the positivity of sentinel lymph note was lower (17.6% vs. 25.7%), when compared to the rest of the cohort. The study published in 2023 by the Italian team, aiming to strictly follow the characteristics of patients with melanoma of the head and neck and indication for SLNB, had a total number of 93 cases in the period 2015–2021 with an average age of 58 years (50–70) and demonstrated a positivity rate of 19.35% with a Breslow index (2.2 mm; 1.8–5.0 mm), higher than the negative ones (1.8 mm; 1.1–3.0 mm) [40]. In another study, conducted by Quaglio et al., the rate of positive SLN was just slightly different from our results (25.0%) [41]. In this study, the authors also analyzed the potential predictivity of micro–macro–metastatic pattern, Breslow index, and ulceration, and the results showed that there is a higher likelihood of a non-sentinel lymph node for all of these factors.

In comparison, our results revealed similar age tendency and frequency of positive SLN, but a greater average of Breslow index for patients with head and neck melanoma, the variations could be attributed to the different numbers of subjects examined.

As for lymphoscintigraphy, measured radioactivity in our study showed that for the inguinal basin, the radioactivity captured by lymph nodes was higher than the other regions, data in accordance with one study, which shows that the detection after the radioactive load on lymphoscintigraphy is the highest in inguinal–femoral and axillary lymphatic basins and low in cervical region [28,42]. Meanwhile, there is no noticeable association between positive SLN and radioactivity in our study. Despite all that, the literature’s data are insufficient regarding the research on the relation between melanoma [43] and lymphoscintigraphic investigation of the sentinel lymph node [12,44], therefore, it might be worthwhile to perform a more thorough analysis in this regard.

The first most significant limitation of this study is that it was conducted in a single center. As a second limitation is the number of patients enrolled, SLNB technique with lymphoscintigraphy being a procedure available only in a few medical centers in Romania. Third, only a low number of patients had the BRAF gene tested, and no gene subtypes were examined; this information might have some bearing on the connection with SLN positivity.

## 5. Conclusions

The average age at diagnosis of malignant melanoma was 54.3 ± 14.4 years, with advanced stages of the disease (>50% stage III, IV), an average Breslow index of 3.04 ± 2.8, and a sentinel lymph node positivity rate of 24.6%.

Preoperative nodes’ radioactivity varied depending on the lymphatic drainage basin, with the inguinal nodes having the highest load (*p* = 0.015). There was no statistically significant association between the radiotracer load and the positivity of the lymph node.

The elective region of melanoma localization for patients 70 years or older is head and neck (*p* = 0.007), advanced stages are more frequently identified (*p* = 0.044), the positivity rate of SLN is higher (*p* = 0.045), and complications during surgery are more common (*p* = 0.004).

## Figures and Tables

**Table 1:** General and surgical characteristics of patients.

Cohort Characteristics	N = 122
Age (years) mean ± SD	54.3 ± 14.4
Lymph node removed n, %	
1	84, 68.9%
>1	38, 31.1%
Positive SLN n, %	30, 24.6%
Male n, %	62, 50.8%
Menopause N = 60, n,%	37, 61.6%
Melanoma site	
Head and neck n,%	17, 13.9%
Thorax n,%	39, 32.0%
Abdominal n,%	18, 14.8%
Upper limbs n,%	21, 17.2%
Lower limbs n,%	27, 22.1%
pT3 and pT4 stage n,%	61, 50.0%
Breslow index mean ± SD	3.04 ± 2.8
Oncological safety margins (mm)	
1	35, 28.7%
1.5	3, 2.5%
2	84, 68.9%
Period from diagnosis to surgery (days) mean ± SD	30.3 ± 5.1
BRAF gene, N = 31 n,%	22, 70.9%
History of cancer n,%	5, 4.1%
Body mass index (kg/m^2^) mean ± SD	24.1 ± 2.7
Type anesthesia n,%	
Local	30, 25.6%
General	92, 75.4%
Cardiovascular diseases n,%	39, 31.9%
Surgery complications n,%	7, 5.7%
Surgery’s duration (min) mean ± SD	125.0 ± 27.9
Reintervention n,%	2, 1.6%
Seroma n,%	18, 14.8%
Prophylactic antibiotic therapy n,%	119, 97.5%
Postoperative antibiotic therapy n,%	119, 97.5%
Duration of antibiotic therapy	3.8 ± 1.4
Preoperative anticoagulant therapy	6, 4.9%
Preoperative antiaggregant therapy	8, 6.6%
Switching anticoagulant therapy	6, 4.9%
Wound healing (days) mean ± SD	12.5 ± 1.9

**Table 3:** Comparison depending on age.

	<70 Years Old	>=70 Years Old	*p*-Value
	N = 97	N = 25	
Lymph node removed n, %			0.918
1	67, 69.1%	17, 68.0%	
>1	30, 30.9%	8, 32.0%	
Positive SLN n, %	20, 20.6%	10, 40.0%	0.045
Male n, %	51, 52.6%	11, 44.0%	0.444
Melanoma site			
Head and neck n,%	9, 9.3%	8, 32.0%	0.007
Thorax, n,%	33, 34.0%	6, 24.0%	0.338
Abdominal n,%	13, 13.4%	5, 20.0%	0.407
Upper limbs n,%	19, 19.6%	2, 8.0%	0.171
Lower limbs n,%	23, 23.7%	4, 16.0%	0.408
pT3 and pT4 stage n,%	44, 45.4%	17, 68.0%	0.044
Breslow depth mean ± SD	2.82 ± 2.5	3.88 ± 3.5	0.079
Period from diagnosis to surgery (days) mean ± SD	30.4 ± 5.1	29.9 ± 5.4	0.715
BRAF gene, N = 31 n,%	16 from 22	6 from 9	0.736
History of cancer n,%	4, 4.1%	1, 4.0%	1.000
Body mass index (kg/m^2^) mean ± SD	20.21 ± 2.8	23.84 ± 2.7	0.536
Type anesthesia n,%			0.550
Local	25, 25.8%	5, 20.0%	
General	72, 74.2%	20, 80.0%	
Cardiovascular diseases n,%	22, 22.7%	17, 68.0%	<0.001
Surgery complications n,%	2, 2.1%	5, 20.0%	0.004
Surgery’s duration (min) mean ± SD	123.30 ± 26.9	131.6 ± 31.4	0.082
Reintervention n,%	0	2, 8.0%	0.041
Seroma n,%	12, 12.4%	6, 24.0%	0.202
Postoperative antibiotic therapy n,%	123.30 ± 26.9	131.6 ± 31.4	0.082
Duration of antibiotic therapy	3.77 ± 1.4	3.84 ± 1.1	0.903
Preoperative anticoagulant therapy	1, 1.0%	5, 20.0%	0.001
Preoperative antiaggregant therapy	4, 4.1%	4, 16.0%	0.055
Switching anticoagulant therapy	1, 1.0%	5, 20.0%	0.001
Wound healing (days) mean ± SD	12.22 ± 1.8	13.48 ± 2.1	0.004

## Data Availability

The data presented in this study are available in the article.
